# What Veterinarians Need to Know About the Newly-Emerging Field of Insects-as-Food-and-Feed

**DOI:** 10.3390/vetsci12010012

**Published:** 2024-12-31

**Authors:** Kimberly L. Boykin, Mark A. Mitchell

**Affiliations:** Department of Veterinary Clinical Sciences, Louisiana State University, Skip Bertman Dr, Baton Rouge, LA 70803, USA

**Keywords:** insect farming, epidemiology, biosecurity, food safety, insect immunology

## Abstract

Over the last two decades, the insects-as-food-and-feed industry has rapidly emerged. Its growth is largely because insects require substantially less resources (water, food, and energy) to produce than traditional sources of animal protein, making it a sustainable alternative food option. As this industry continues to grow, veterinarians will likely be called upon to assist in identifying food safety concerns, assessing animal health, implementing biosecurity measures, and formulating/prescribing treatment protocols comparable to what we have seen with the honeybee industry and the institution of veterinary feed directives (VFDs). Similar to other agricultural markets, high animal densities and management practices put insects at high risk for infectious diseases. Veterinarians interested in working with these species will need to become knowledgeable regarding the diseases afflicting the feeder insect industry and how best to diagnose and treat pathogens of concern. Using the edible cricket industry as an example, this review will highlight health and production issues while drawing similarities to other traditional livestock operations. If the insects-as-feed-and-food industry is going to be viable, veterinary involvement will be essential to ensure that insects can be used as a safe source of food for all.

## Simple Summary

As the world’s population continues to expand, it will be important for us to find new ways to ensure that high quality animal protein is available to meet the needs of this growing population. Insects as feed or food for animals and humans, respectively, is one source of animal protein that is underutilized but could help meet this demand, while reducing the impacts on the environment. While there is a great deal of interest in growing insects, or mini-livestock, for feed and food, there has been minimal study into the diseases of these organisms under production conditions. The purpose of this review is to share how veterinarians, the individuals charged with managing and controlling disease in livestock populations for aquaculture, poultry, swine, caprine, and cattle, can serve the same role for insect production.

## 1. The Need to Feed an Ever-Growing Population

### 1.1. Global Food Insecurity and Malnutrition Is a Public Health Threat

The world population currently stands at 8 billion people, and current projections expect the population to rise to 10 billion people by 2050 [1]. As the population grows, so does our need for more food, water, and other natural resources. However, the question remains about whether our planet is capable of supporting this amount of growth. Even at our current numbers, we have major shortcomings resulting in insufficient access to healthy foods. In the latest set of statistics released by the Food and Agricultural Organization (FAO), it has been estimated that 9.2% of the world’s population is undernourished, which is a 1.3% increase from 2019 and represents an additional 100 million undernourished people over a four-year period [2]. Global food insecurity and malnutrition are public health threats that must be addressed. Malnutrition is the single largest contributor to disease and is the number one cause of death in children under the age of five [2]. If we continue to produce and distribute food using our current traditional systems, those numbers will continue to increase.

### 1.2. Traditional Livestock Systems Are Not Sustainable

Malnutrition and lack of food are not the only concerns associated with an ever-increasing human population. Even without the extra food burden of two billion people, our current systems and traditional livestock operations are unsustainable and damaging to the environment. Forty-five percent of the world’s surface is already dedicated to sustaining agricultural production, with one-third being used as cropland for both human and animal consumption and two-thirds being utilized for livestock grazing [3,4]. The livestock industry is responsible for 15% of all global greenhouse emissions, second only to the energy sector [5]. Livestock production is also a massive contributor to deforestation, soil erosion and degradation, loss of biodiversity, and water pollution [6]. Yet in developed countries, the demand for animal protein has continued to increase. Current estimates have shown that the demand for ruminant meat alone is projected to increase 88% between 2010 and 2050 [7]. Even if crop yields continue to increase at their current rates, it is estimated that an additional 593 million hectares would be needed for agricultural land, which is nearly twice the size of India [8]. The demand for animal protein is not limited to livestock. Over the past 50 years, global production of fish and seafood has quadrupled, putting unprecedented pressures on the global fish stocks and the oceans’ ecosystems [9]. Drastic shifts in the production and consumption of animal protein are needed to limit the negative effects of our current food systems.

### 1.3. Entomophagy as a Sustainable Solution to Food Insecurity

One proposed solution to the world’s food insecurity and environmental issues is entomophagy, or the consumption of insects [10,11]. Entomophagy is not a new concept. Humans have been eating insects for centuries, and many people around the world incorporate insects into their daily diet [11,12]. However, scientists and Western cultures have only recently started evaluating this practice to support growing populations in a more sustainable way, either by putting them into animal feeds as a way to reduce our reliance on soybean and fish meal ingredients, or by raising them purely for human consumption [10,11]. Insect farming, also referred to as mini-livestock farming, has several key advantages over traditional livestock operations. Depending on the scale of the operation, insects can be very easy to raise in captivity and require minimal economic investment to get started. Moreover, insects can be raised virtually anywhere in the world, including in dense urban areas, and need very little land space to be grown because they can be grown in vertical spaces. Localizing food production to urban centers has the potential to decrease the need for transportation and could lower gas emissions [10,11,13]. Lastly, and most importantly, insects require less feed and water input than traditional livestock due to their high feed conversion efficiency. Table 1 highlights the amount of feed that is required to raise one kilogram of edible meat for crickets versus the more traditional livestock animals [10]. Each of the traditional livestock species requires two to twelve times more feed due to their endothermic nature and requirement for more energy input and because the whole animal is not consumed (e.g., bones and certain organ meats are often not eaten by human consumers). Additionally, certain insects (black soldier fly [BSF] larvae [*Hermetia illucens*]) can even be grown on waste materials, including manure, when not being used for human consumption. These facts are the driving force behind using mini-livestock to replace some of our more traditional protein sources in animal and human foods.

## 2. The Current State of the Feeder Insect Market

### 2.1. Insects as Feed and Food

The current insect industry is divided into two parts: insects being utilized in or as animal feeds (insects as feed) and insects being utilized for human consumption (insects as food). Insects are a high-quality nutrition source that are high in protein and several different vitamins and minerals [14]. Combine this with the environmental benefits over traditional livestock that were mentioned previously, and the high prevalence of certain insects, namely BSF larvae, around swine and poultry farms, and it did not take long for researchers to begin experimenting with using BSF larvae in various agricultural feeds. Over the last decade, research into BSF larvae as an ingredient in animal feeds has risen exponentially, and it is now an Association of American Feed Control Officials (AAFCO)-certified ingredient that can be used in feedstuffs intended for salmonoids, poultry, swine, and adult dogs [15]. Other insects continue to be researched as an ingredient as well, but as of this article have not been officially approved by AAFCO for use in the United States. In Europe, eight insect species (BSF larvae, common housefly larvae [*Musca domestica*], yellow mealworm larvae [*Tenebrio molitor*], lesser mealworm larvae [*Alphitobius diaperinus*], house crickets [*Acheta domesticus*], banded crickets [*Gryllodes sigillatus*], field crickets [*Gryllus assimilis*], and silkworms [*Bombyx mori*]) have been approved in some form or another to be used in most agricultural and pet feeds [16]. Even if insect consumption does not become mainstream for humans, incorporating more energy-efficient ingredients into animal feeds will at least help offset the impacts caused by increased numbers of livestock and companion animals.

As mentioned previously, entomophagy is well accepted outside of Western cultures, and over the last decade, we have seen an increase in demand for insect-based proteins within the Western world too. As an example of this, in 2023, the edible insect market was estimated at 3.2 billion USD; however, it is expected to grow to 7.6 billion USD over the next five years [17]. Although demand is still low, consumer acceptance continues to increase, especially for products that disguise insect protein using insect meals and flour instead of using whole insects [18]. Aside from the disgust factor, one of the biggest hurdles to overcome regarding consumer acceptance is the currently high prices associated with human-grade food insects. As of writing this article, cricket powder is currently selling for $1.87–4.28 per ounce [19], while a pound of ground beef is selling at $5.22 (or 0.33 cents per ounce) [20]. In our current economy, fewer people are going to select the more sustainable option if it is, at a minimum, five times more expensive than the familiar and well-accepted option. Consumer research into the organic food industry has shown that many people are not willing to pay a premium of more than 30 percent for organic food [21]. Although not quite the same, the organic food industry and mini-livestock industry are both niche markets that appeal to people wanting to make healthier and greener choices about the origin of their food, so we should expect similar levels of price premiums to be found acceptable. As the industry continues to grow and improve in efficiency, the price of insect products should decrease; however, a lot of work still needs to be conducted to achieve these comparable price points.

### 2.2. Current Limitations to the Feeder Insect Market

The insects-as-food-and-feed industry is still relatively new, and as such, there are still a few areas of concern that must be addressed in the coming years to ensure the industry’s success and survival. These include, but are not limited to, further research into combating insect disease and production losses, insect escapes, food safety concerns, and how best to regulate the industry to ensure that a safe product is consistently being produced.

#### 2.2.1. Low Production Numbers

One of the biggest issues facing the commercial insect industry at this time is poor production numbers, and these can be directly correlated to the high prices associated with current insect production. Mass-reared insects have been plagued with viral, bacterial, fungal, and parasitic diseases for several decades now, with some diseases, particularly viruses, capable of causing 100% mortality. Several review articles have already been published discussing the major diseases within the industry and are outside the scope of this review [22,23,24,25]. Out of the major mass-reared insect species, only BSF larvae have shown themselves to be resistant to most pathogens; however, that could change quickly as new pathogens can emerge every day, especially under the high density/high stress environments that mass-reared insects are exposed to. As of now, very little is known about the epidemiology of diseases affecting mass-reared insects. To reduce the burden of these diseases, more research is needed to understand the prevalence of these diseases, how they are transmitted, how the diseases progress in individual insects, and what can be done to mitigate the spread and severity of disease. Evidence-based research has led to improvements in veterinary medicine and thus our management of diseases in traditional livestock systems that often contend with the same high density/high stress environments. Advances in diagnostic technologies have allowed for better screening and confirmatory tests for diseases of industry concern. Vaccines and improved medical treatments have allowed for reductions to morbidity and mortality. Changes to biosecurity and animal management have been used to reduce the spread of diseases between farms and between age groups. The application of these same types of epidemiological evidence-based methods are needed to improve insect production systems and ensure their long-term success and sustainability.

Other areas of mini-livestock production that would also benefit from additional research include nutrition and genetics. Enhancements in these areas could lead to higher yields and genetic lines that are larger and hardier against disease. Luckily, the insect industry has the benefit of decades of scientific knowledge from the traditional livestock industries regarding genetics, selective breeding, and nutrition to increase their production numbers. The livestock industry as we know it is hundreds of years old, but thanks to advances in medicine and genetics we are still seeing improvements to this day. In just the last twenty years, chickens have increased egg laying rates by 13.6% and broilers have increased in weight by 23.9% [26]. Turkeys, cattle, and hogs have increased in weight by 20.5%, 10%, and 9%, respectively, during that same time [27]. For the insect industry, improving genetic lineages has already shown some improvement in yields for BSF larvae [28]. As the industry and genetic lines change, we may see that additional tweaks to our current husbandry and diets are needed as well, to continue to support health and growth.

#### 2.2.2. Food Safety Concerns

Insects are often touted as a low-pathogen food; however, more evidence-based research needs to be done to address food safety concerns. Previous literature has shown that insects can harbor several pathogens of concern, including *Clostridium* spp., *Campylobacter* spp., potentially pathogenic members of the Enterobacteriaceae family, and spoilage-related *Pseudomonas* spp. [29,30,31]. Additionally, feeder insects have been analyzed for the presence of antibiotic resistance genes and have been found to harbor genes important to tetracycline and macrolide resistance [31]. Animal products and meat are an important source to consider when discussing the transfer of antibiotic resistance genes between species. Once bacteria harboring resistance genes enter the gastrointestinal tract, those genes may be transferred to our own microbial community, making our more common antibiotic choices less efficient in controlling infections as they arise. Processing methods such as oven-drying and boiling can help with reducing the bacterial load and may damage DNA associated with antibiotic resistance genes, but concern should still lie with bacteria that produce heat-resistant spores or inefficient heating processes. Another concern is with freeze-drying insects. This is a common method of preparing whole insects for human and animal consumption, and even though it reduces the water content of the food, and therefore the ability of bacteria to grow on the food items, it does not kill bacteria that is already present, especially those residing within the gastrointestinal tract of the insect [32].

Other food safety concerns are associated with the exposure to toxins and other chemicals that insects may bioaccumulate throughout their lifetime or produce as part of a defense mechanism. Even insects grown under controlled conditions may be exposed to pesticides, fertilizers, and drug residues that could bioaccumulate and be transferred through the food chain. Heavy metals are of particular concern with feeder insects. Many edible insects grown under standard rearing conditions have been found to contain toxic concentrations of heavy metals, including copper, zinc, iron, lead, cadmium, mercury, and arsenic [33]. Further research is needed to ensure that we are limiting the effects of these toxins and that only safe levels are being accepted into animal feedstuffs and human foods, similar to traditional livestock species.

The final food safety concern to address is related to food allergies. Preliminary research has suggested that individuals with sensitivity to shellfish-type foods may also be sensitive to certain chemicals found in insects. Common allergens found in invertebrates include tropomyosin, arginine kinase, and glyceraldehyde 3-phosphate dehydrogenase (GAPDH), which have a substantial amount of cross-reactivity within the invertebrate class [34]. However, some people with known shellfish allergies have been able to ingest insect proteins without issue, and those with no known allergies have reacted negatively to insect proteins. Not all allergic reactions have occurred after ingestion, either. Some reactions have been caused by inhalation or direct contact, making those working with insect products and their close contacts at risk as well. More research is needed to identify the exact major and minor allergens of concern in insects to determine if anything can be done to limit their immunoreactivity with post-processing methodologies. In the meantime, foods containing insect ingredients should be well labelled to ensure that consumers are aware of the potential allergenic risk of the product. Based on all of these important food safety aspects, having more government regulation and oversight is necessary, especially since the industry is in need of more research to fully understand all of the risks associated with it. It would be prudent of the United States government to be more proactive and cautious considering there are still so many unknowns related to the industry.

#### 2.2.3. Government Regulations

As of now, the United States Food and Drug Administration (FDA) regulates both feed and food aspects of the industry. Insects are more commonly being used as ingredients in animal feeds, usually in the form of insect meal. Animal feeds must use ingredients that have received approval from AAFCO or that are “Generally Recognized as Safe (GRAS)”. Only BSF have been approved as a feed ingredient by AAFCO. Insects are not currently listed on the GRAS approved list; however, ingredients that are “obviously safe” do not have to be included on the GRAS list. This has led to some confusion within the industry as to whether any insects besides BSF can be used. A push for further guidance from the FDA regarding insect ingredients is warranted. As for human consumption, the FDA has stated that insects can be used as an ingredient if the insects are “clean and wholesome (i.e., free from filth, pathogens, toxins); must have been produced, packaged, stored, and transported under sanitary conditions; and must be properly labeled (with use of scientific name)”. They also specify that insects “must be raised specifically for human food following good manufacturing practices” and that they cannot be diverted from the pet food industry or collected from the wild [35]. However, every other live animal industry (e.g., meat, poultry, eggs, and seafood) is regularly inspected by either the FDA or USDA. Guidance documents also exist for each industry that address specific concerns to ensure wholesome food [36]. Having more oversight by government agencies would help to ensure a cleaner, safer product and may help consumers feel more at ease about trying this novel source of protein.

## 3. The Cricket Industry

### 3.1. Life Cycle and Uses

**Figure 1:**
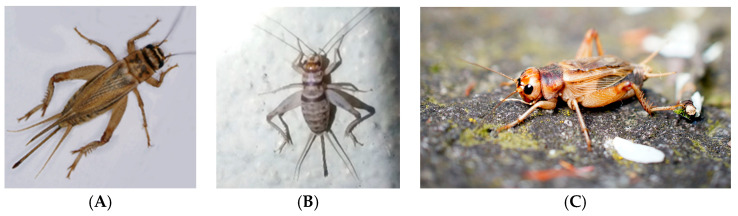
Visual representations of the top three cricket species used in the feeder insect industry, (**A**) *Acheta domesticus* cricket, adult female [40]; (**B**) *Gryllodes sigillatus* cricket, subadult female [41]; (**C**) *Gryllus assimilis* cricket, adult male [42].

Crickets are one of the top three insects being used in the insects-as-food-and-feed industry [37,38,39]. Currently, crickets are primarily grown as live feeder insects for captive reptiles and other insectivores [38]. However, cricket meal is becoming more popular to use as a high protein ingredient, particularly when added to flour for use in different forms of baked goods, chips, and protein bars [38]. The top cricket species used in the industry are the European house cricket (*Acheta domesticus*), banded cricket (*Gryllodes sigillatus*), and Jamaican field cricket (*Gryllus assimilis*) (see Figure 1) [39].

Under normal rearing conditions, *Acheta domesticus* crickets have an approximate 9-week life span. Adults lay eggs in soil pans that can be removed and placed in their own bin away from the adults. Nymphs emerge after a 2-week incubation, and then molt into larger and larger sizes over their entire life cycle. At approximately 6 weeks old, they molt into an adult, become sexually mature, and lay eggs over the course of one to two weeks [43]. Overall, crickets are easy to raise, needing very little care, but there are points within the life cycle where husbandry and biosecurity could be improved to provide higher yields.

### 3.2. Significant Morbidity and Mortality Concerns

The cricket industry is one of the most impacted by high production losses. Some producers consistently see losses >50% over the course of the life cycle [44]. This results in a loss of economic revenue and any resources consumed during early development periods. Some of the losses can be attributed to issues with husbandry. Eggs and young nymphs are very sensitive to temperature and humidity levels [45,46,47]. These factors may also play a role in disease transmission and propagation in older nymphs, so further research into these factors should be a priority [48,49]. Young nymphs are also very prone to starvation and dehydration if they are unable to locate their food and water sources or drown in water sources that are too deep for their size. New innovations with bin setup are helping to control some of these losses. Specific diet formulations can also be created to help support health and immunity instead of just growth.

While diet and husbandry are important, disease transmission is often the biggest cause of morbidity and mortality in mass-reared crickets [22,23,24,25]. To meet production needs and to counteract the high losses seen during their 9-week life cycle, crickets are often reared in high density situations with thousands of crickets in a single bin. The bin sizes and cricket densities vary by producer, and there is limited evidence-based research characterizing the best practices for cricket rearing. These high densities and the close quarters make it easier to transmit diseases between crickets [22,23,24,25,43,48]. Combine this with poor biosecurity, compared to other livestock industries, and we can see why infectious diseases are of great concern.

In many facilities, all age groups are raised in the same building, allowing older cricket stocks to continuously infect the younger generations [50]. In other industries, particularly poultry, different age groups are kept separate in an all-in–all-out practice that limits spread between generations. This type of set up may be more challenging to do with crickets, since they are not always harvested at a set age or weight. Live-feeder crickets are often harvested at younger ages to feed smaller animals; while others of the same age are left behind to ensure a continuous stock of each age group, leading to underutilized space within buildings as the crickets age [50]. An all-in–all-out practice would be much easier to maintain in a facility focused on producing a single age of cricket, as would be the case for crickets used for ingredients only. The other benefits of an all-in–all-out practice are that it allows for deep cleaning and disinfection of the facilities between age groups and less cross-contamination happening through workers and fomite spread [51]. When all ages are raised at the same time, it becomes much more difficult to fully clean building spaces, and incidental spread through workers and tools is much more likely to occur. A cost–benefit analysis would be needed to determine if the extra cost needed to maintain this type of setup would be effective enough to justify the production gains.

Other biosecurity procedures that could be applied to the cricket industry that are often not followed include better facility entrance protocols with vehicle disinfection, limiting deliveries, and restricting non-workers from entering the facility. Buffer zones could be added around insect buildings, and workers could change footwear and clothing and/or shower in and out upon entry and exit of the facility. Additionally, tougher pest control measures should be implemented, and only one insect species should be raised at an individual facility. Although drastic, these types of changes have made quite a difference in disease control for other livestock industries, particularly poultry houses [52].

Ideally, a farm should not incorporate additional insect stocks from other farms, but under certain circumstances, particularly if needing to improve genetic lineages, such a transfer may be necessary. Under such conditions, strict quarantine and disease screening should be implemented to ensure that no new pathogens are being introduced to the farm. An offsite location should be used for the testing prior to transporting them to the primary farm site. The insect industry also needs to identify ways to recognize sick (and dead) insects within a bin and remove them from the rest of the population in order to reduce the spread of disease. This is particularly important for cricket populations that are prone to cannibalism of conspecifics, which is known to spread certain viruses throughout the population [22,25].

## 4. Cricket Diseases and Parallels to Traditional Livestock Diseases

Using the *Acheta domesticus* cricket industry as an example, we will review some key pathogens that have greatly affected the industry and how to better control these pathogens using techniques that traditional livestock operations have used to control their own pathogens of concern.

### 4.1. Acheta domesticus Densovirus (AdDV)

First, we will look at *Acheta domesticus* densovirus and its effects on the cricket industry. AdDV is a member of the *Parvoviridae* family. Like other members of this family, it is a small, non-enveloped virus that is quite hardy and resistant to several different methods of disinfection. It can persist in an environment for a long period of time and mostly spreads through fecal–oral transmission, although airborne transmission has not been ruled out as a possibility [53]. AdDV was first identified in 1977 at a Swiss cricket-rearing facility, where it spread rapidly throughout the population and led to an almost 100% mortality rate [54]. Additional worldwide outbreaks have occurred since that time, and it is now thought to be endemic in most commercial populations. The virus has been found to have a tissue tropism for the fat body, midgut, hypodermis, and Malpighian tubules where it causes hypertrophy of the cell nuclei and intranuclear inclusion bodies [53,54,55]. Clinical signs often present within the last three nymphal instars or at adult emergence and include anorexia, absent gut motility, retarded growth, sluggishness and reluctance to jump, which progresses to full hindlimb paresis, and eventually death. *Acheta domesticus* crickets are primarily affected by the virus; however, other insect species (*Gryllus assimilis*, *Gryllodes sigillatus*, *Tenebrio molitor*, and *Zophobas morio*) have tested positive despite little to no clinical signs being present. It has not been determined whether the off-target species are capable of supporting active replication of the virus [53,56].

Because most veterinarians are unfamiliar with invertebrate medicine, it can be useful to use examples of pathogens from more familiar species to help veterinarians identify the epidemiological patterns between species to increase their confidence with invertebrates. An example for AdDV is porcine parvovirus (PPV). Both viruses are within the *Parvoviridae* family, and thus share a lot of similarities. PPV is a highly prevalent virus and is endemic in most swine herds worldwide. It is one of the major causes of reproductive failure in swine and is associated with stillbirths, mummifications, embryonic death, and infertility (SMEDI). It has proven difficult to completely disinfect facilities infected with PPV, and control of the virus has instead been focused on vaccination and natural exposure of naïve adult pigs prior to gestation. What can this virus teach us about AdDV? First, we likely will not be able to eradicate AdDV from commercial cricket facilities. The virus is too hardy and ubiquitous in the environment to eliminate it, so re-infection would be too likely of an outcome [57,58]. Our efforts will probably be better spent working towards finding more resistant crickets and developing molecular technologies to support the cricket’s immune system since vaccination (in the vertebrate sense of the word) is not possible. This may involve using emerging RNAi technology, some form of immune priming against the virus, or molecules such as antimicrobial peptides or probiotics to help bolster the cricket’s immune system. Second, if we do find a workable solution for controlling the virus, we should not become complacent with our management techniques or fail to continue research into the subject. Over the last two decades, PPV has continued to produce strains that cannot be neutralized by older vaccines. As with many viruses, it continues to evolve, and new variants and new viruses have been identified that continue to challenge the porcine industry [59].

### 4.2. The Challenge of Multi-Factorial Disease Complexes

AdDV is one of the better-studied viruses affecting the cricket industry; however, we know of at least nine other viruses that have been identified in *Acheta domesticus* cricket stocks alone [60]. How much of a role these viruses play in production losses has yet to be determined. For some of these viruses, researchers have not yet determined whether they result in clinical disease. Even less is known about bacterial, fungal, protozoal, and parasitic pathogens of crickets. Future epidemiological research should focus on identifying all possible pathogens of concern, determining the prevalence of each of these pathogens, how they are transmitted and their pathogenicity risk, and how common co-infection is and whether it impacts severity of disease.

We know from traditional livestock medicine that many disease processes are caused by complex, multi-factorial interactions. Take for instance shipping fever in cattle, also known as bovine respiratory disease complex (BRCD). This respiratory disease is known to be caused by a combination of different factors including environmental stressors, host immunity and susceptibility, and the presence of certain pathogens. Usually, it involves initial infection with a viral pathogen or multiple stressors that alter the cow’s defense mechanisms and allows for colonization of the lower respiratory tract with an opportunistic bacterial agent. Stress factors can include transport, inclement weather, crowding, and/or inadequate ventilation [61]. Mass-reared crickets are often subjected to multiple stressors as well, particularly high densities, access to lower quality foodstuffs, poor ventilation, and inadequate temperature or humidity levels. Until we know more about the epidemiology of cricket diseases, it would be fair to assume that some of the losses we are seeing clinically are much more complex than just a single virus. As such, we should be attempting to lower the impact of environmental stressors by improving rearing conditions and practicing strict biosecurity to try and limit the number of co-infections capable of causing disease.

## 5. Treatment of Diseases in Mini-Livestock

### 5.1. The Invertebrate Immune System

Creating a vaccine to protect against insect pathogens is not as straightforward as it is for vertebrate animals. Insects lack antibodies, thus making traditional vaccines ineffective [62]. They do, however, have other methods of protecting themselves against pathogens. While we do not have a full comprehensive understanding of the invertebrate immune system, we do understand several of its components and how certain pathways work together to fight different types of pathogens.

Insects possess pattern recognition receptors on many different types of cells capable of recognizing pathogen-associated molecular patterns or PAMPs. Recognition of a PAMP activates multiple immune signaling pathways that are analogous to the vertebrate signaling pathways of Toll, Imd (TNF-α), and Jak/Stat. These pathways result in changes to cellular gene expression and amplify the immune response, leading to activation of the humoral and cellular effector responses [62,63,64,65,66,67,68,69].

In the humoral response pathway, certain chemicals are released that help to break down cell walls and weaken pathogens. These chemicals can include antimicrobial peptides (AMPs) such as lysozyme and defensins that directly damage the cell wall, or it can include complex enzyme cascades like the prophenoloxidase system that is responsible for regulating the melanization process that involves toxic and reactive intermediates to destroy foreign cells [63,64,70,71].

In the cellular response pathway, we see insect hemocytes utilizing phagocytosis, encapsulation, and nodulation to trap and destroy foreign cells, parasites, and debris. Encapsulation and nodulation are similar processes to granuloma formation, where foreign material is covered in layers of granulocytes and plasmacytes and walled off from the rest of the body. Encapsulation usually involves foreign material that is too large to be phagocytosed (e.g., parasites) and may or may not involve additional humoral processes like melanization. Nodulation is usually associated with large aggregates of bacteria or fungi and always involves melanization of the material [63,64,70,71].

Although insects lack antibodies, some species have been shown to have some degree of “memory” regarding mounting a better/faster immune response to pathogens they have been exposed to previously. There have also been instances of insects being capable of passing this “memory” on to offspring. This phenomenon has been termed immune priming. Different mechanisms of immune priming may exist, but one such mechanism that has been studied involves mothers passing along PAMPs to offspring via the vitellogenin of developing eggs [72]. This process allows young insects to have more protection against common environmental pathogens during life stages where they may be more immunocompromised. Learning more about this system in feeder insects may allow us to exploit this process to better protect insects from common pathogens encountered in a commercial setting.

For viral pathogens, insects can utilize some of the pathways already mentioned, but the most robust system for neutralizing viruses is the RNA interference (RNAi) pathway; this system has been well conserved throughout evolution from prokaryotes to eukaryotes. The process recognizes double stranded RNA (dsRNA) molecules which are utilized by certain viruses as a replication intermediate and not often found within the host naturally. Once the host recognizes these dsRNA molecules, it creates small RNA strands (silencing RNA or siRNA) that are complementary to the target strand. These siRNA then bind to Argonaut family proteins and trigger the degradation of longer target RNA strands [73]. Although it is thought that RNAi initially evolved as an antiviral immune response, other pathways have also been found in insects to regulate gene expression (microRNA or miRNA) and suppress germline transposon expression (Piwi-interacting RNA or piRNA) [74]. While RNAi is a highly efficient and systemic system in some insect orders (Coleoptera), it does appear to be variable and inefficient in others (Lepidoptera, Diptera, Hymenoptera, and Hemiptera) [75].

With antibiotic resistance on the rise and an ethical dilemma over whether insects should receive antibiotic therapy for infections, it would be prudent for those working in this field to start identifying how best to utilize the insect’s own immune system to fight off infections. This may involve improving genetic lineages, manipulating gene expression, or utilizing AMPs, immune priming, or RNAi as alternative treatments or preventatives.

Several clinical trials are already underway in human and veterinary medicine to determine the safety and efficacy of AMPs to treat certain conditions [76]. Quite a bit of work needs to be done in this field to determine whether the use of exogenous or indigenously stimulated AMPs would be worth the cost associated with providing them to feeder insects. Studies would also need to be conducted to determine which conditions and which AMPs are most suitable to use, and how best to administer or stimulate their production.

The premise of immune priming has made tremendous progress in the honeybee industry. As of January 2023, the USDA has conditionally approved the first-ever “vaccine” for insects. The vaccine produced by Dalan Animal Health, Inc. is meant to be used in *Apis mellifera* honeybees to protect hives against American foulbrood, which is caused by the spore-forming bacterium *Paenibacillus larvae*. The premise of the vaccine is to provide killed whole-cell *P. larvae* bacteria orally to worker bees. They then incorporate the vaccine into royal jelly that is fed to the queen, and fragments of the bacteria are deposited into her ovaries, providing transgenerational immune priming to the queen’s offspring [77]. While the premise appears to work for honeybees, more research is needed to determine if a similar method could be developed for other insect species.

Finally, RNAi technology has the greatest potential for helping us to manage viral diseases of insects. Despite having a variable efficacy in certain insect orders and species, the Coleopterans, which comprise a moderate portion of the feeder insect market with *Tenebrio molitor* and *Zophobas mori*, have been found to have a robust and highly efficient RNAi pathway. However, most of the studies performed in Coleopterans have been aimed at developing technology for pest control purposes rather than viral control to improve insect health. Further research needs to be conducted to ensure that the technology is safe and effective for that purpose. As for Orthopterans, not much work has been done, but a transcriptomic analysis of the *Acheta domesticus* genome has shown that all components for a robust RNAi system are present [78]. Another study used *Acheta domesticus* crickets as a model organism for *Penaeus merguiensis* (prawns) infected with densovirus and showed that orally administered dsRNA could be used to stimulate the RNAi system and had fairly good results with increasing survival times [79]. This study encourages further research to determine if AdDV or other viruses of concern could also be managed with the use of RNAi technology.

### 5.2. The Insect Microbiome and the Role of Probiotics

Exploration into the insect microbiome has exploded over the last several years. While the true microbiome encompasses bacteria, archaea, viruses, and eukaryotes, for the purposes of this short review, the focus will only be on the bacterial microbiome. Multiple studies have been published for the major feeder insect species that have worked to identify dominant bacterial phyla, possible foodborne pathogens, and how diet and environment may alter the proportions of bacterial families that are present [29,30,31,80,81,82,83,84,85,86,87,88,89,90,91]. Across many insect orders, Proteobacteria, Firmicutes, and Bacteroidetes tend to be the three most dominant phyla represented, and this appears to hold true for the feeder insect species as well. Knowing more about the insect’s microbiome is important as we now know that these organisms can have wide-reaching, whole-body effects that impact metabolic, behavioral, and immune functions [92,93].

The species present in the gut microbiome are directly or indirectly involved in many processes including host metabolism, provision of nutrients, degradation of harmful substances, support of reproductive fitness, promotion of host growth and development, and regulation of immune formation and function [94]. Many regulatory mechanisms between the host and normal microbiota exist to maintain the delicate balance of microbial homeostasis. However, diet, environmental conditions, presence of pathogenic organisms, and stress can directly affect the composition of microbes, leading to either the loss of beneficial bacteria and/or the overgrowth of pathogenic bacteria, also known as dysbiosis. Dysbiosis directly affects nutrient availability such as altered ratios of short chain fatty acids, alters the production of certain molecules such as antimicrobial peptides and cytokines, and can dysregulate host transcription and cell turnover rates [95]. Multiple studies have concluded that dysbiosis in insects is often associated with increased disease incidence and shorter life spans [96,97,98].

Therefore, learning how to better support microbial homeostasis should be a priority for the feeder insect industry. As was previously mentioned, diet, environment, and stress can be key drivers of microbial alterations; these factors further reinforce the need to identify optimal diet compositions, best practices for animal husbandry and welfare, and lowering density levels. Another method to support “healthy guts” may be the addition of pre-, pro-, or postbiotics to insect diets. Previous studies in insects have produced mixed results, but most of the research points to these biological feed additives having a beneficial impact on insect health. In honeybees, the addition of *Lactobacillus* and/or *Bifidobacterium* probiotics resulted in lower *Nosema* spore counts [99,100,101] and better resistance to European and American foulbroods [102,103,104]. *Lactobacillus* and *Bifidobacterium* probiotics have also resulted in increased body weights, pupation rates, and silk production in silkworms (*Bombyx mori*), and increased survivability in wax moths (*Galleria mellonella*) [105]. In yellow mealworms, *Pediococcus pentosaceus* and *Enterococcus faecalis* have been shown to reduce mortality and accelerate the rate of larval development [84,106]. In BSF larvae, *Arthrobacter*, *Bacillus*, *Rhodococcus*, and *Saccharomyces* probiotics have all resulted in increased larval rates and/or increased bioconversion rates [107,108,109,110]. Care should be taken when choosing these treatments for each species of insect. While some commercial vertebrate products have produced favorable results, others have led to increased mortality of the host organism and/or reduced larval growth [107,111]. Supplementation with probiotic species that are not normally a component of the host’s microbiome can lead to drastic shifts in the gut flora, causing severe alterations to the host’s normal bodily functions. Pre-screening an insect’s normal microbiome and identifying core bacteria is the best practice for identifying possible probiotic candidates. It is also important to consider the possible effects of a probiotic on human and animal safety as end consumers of the product. All in all, probiotics appear to be of potential benefit to the feeder insect industry, and more research will need to be done to determine the best candidates for specific needs and appropriate dosing strategies.

## 6. Conclusions

The feeder insect industry is continuously growing and will be one of the primary ways we combat food insecurity for our ever-increasing global population. As the industry grows, we will continue to face problems related to viruses and other disease processes that decrease production numbers and increase the cost of producing these insects. To make better management decisions, veterinary- and entomology-focused researchers will need to better define the problems through rigorous epidemiological investigation. This can be done by developing estimates of prevalence, identifying modes of transmission, and determining pathogenicity for each of our pathogens of concern. We will also need to be on the lookout for new and emerging threats to the industry, which can be done by routine screening of insect stocks. Luckily, mini-livestock is coming of age in an era with significant growth in new technologies and medical advances. Treatment options are not limited to traditional vaccines and antibiotics and may also include genetic manipulation, RNAi, antimicrobial peptides, and probiotics. We can also use the lessons learned from traditional livestock medicine to make better management decisions to improve herd health. While feeder insect medicine may seem daunting and unfamiliar, veterinary professionals are well-trained to tackle the issues presented here and should be a valuable resource in helping to develop this industry and elevate it to its full potential.

## Figures and Tables

**Table 1:** Feed conversion efficiency of various livestock organisms [10].

Food Commodity	Kilograms of Feed Required to Produce One Kilogram of Edible Meat
Crickets	2.1
Poultry	4.5
Pork	9.1
Beef	25

## Data Availability

Not applicable.
